# Transcriptome‐based target‐enrichment baits for stony corals (Cnidaria: Anthozoa: Scleractinia)

**DOI:** 10.1111/1755-0998.13150

**Published:** 2020-03-24

**Authors:** Randolph Z. B. Quek, Sudhanshi S. Jain, Mei Lin Neo, Greg W. Rouse, Danwei Huang

**Affiliations:** ^1^ Department of Biological Sciences National University of Singapore Singapore Singapore; ^2^ Tropical Marine Science Institute National University of Singapore Singapore Singapore; ^3^ Scripps Institution of Oceanography University of California San Diego San Diego CA USA

**Keywords:** coral reef, exon, genome sampling, hybrid capture, multilocus data, phylogenomics

## Abstract

Despite the ecological and economic significance of stony corals (Scleractinia), a robust understanding of their phylogeny remains elusive due to patchy taxonomic and genetic sampling, as well as the limited availability of informative markers. To increase the number of genetic loci available for phylogenomic analyses in Scleractinia, we designed 15,919 DNA enrichment baits targeting 605 orthogroups (mean 565 ± *SD* 366 bp) over 1,139 exon regions. A further 236 and 62 barcoding baits were designed for COI and histone H3 genes respectively for quality and contamination checks. Hybrid capture using these baits was performed on 18 coral species spanning the presently understood scleractinian phylogeny, with two corallimorpharians as outgroup. On average, 74% of all loci targeted were successfully captured for each species. Barcoding baits were matched unambiguously to their respective samples and revealed low levels of cross‐contamination in accordance with expectation. We put the data through a series of stringent filtering steps to ensure only scleractinian and phylogenetically informative loci were retained, and the final probe set comprised 13,479 baits, targeting 452 loci (mean 531 ± *SD* 307 bp) across 865 exon regions. Maximum likelihood, Bayesian and species tree analyses recovered maximally supported, topologically congruent trees consistent with previous phylogenomic reconstructions. The phylogenomic method presented here allows for consistent capture of orthologous loci among divergent coral taxa, facilitating the pooling of data from different studies and increasing the phylogenetic sampling of scleractinians in the future.

## 1 INTRODUCTION

Stony corals (Cnidaria: Anthozoa: Scleractinia), numbering over 1,500 extant species, are of great ecological and economic importance, and can be found from shallow waters to great depths across the world's oceans (Cairns, 2007; Huang, 2012; Kitahara, Fukami, Benzoni, & Huang, 2016; Moberg & Folke, 1999). Despite centuries of research into the systematics of Scleractinia (Fukami et al., 2008; Kitahara et al., 2016; Lamarck, 1816; Linnaeus, 1758; Romano & Palumbi, 1996), and even with recent molecular phylogenetic work (Arrigoni, Berumen, Huang, Terraneo, & Benzoni, 2017; Arrigoni, Berumen, et al., 2014; Arrigoni, Terraneo, Galli, & Benzoni, 2014; Huang et al., 2016; Huang, Benzoni, Arrigoni, et al., 2014; Huang, Benzoni, Fukami, et al., 2014; Kitano et al., 2014), the classification and phylogeny of this group remain largely unresolved particularly at the species level (Kitahara et al., 2016). The ideal of a well‐supported and robust phylogeny of corals remains elusive due to several reasons, including inadequate species sampling and the paucity of informative morphological and molecular markers (Budd, Romano, Smith, & Barbeitos, 2010; Kitahara et al., 2016; but see Quattrini et al., 2018).

Since the advent of DNA sequencing, molecular data have been imperative for phylogenetic reconstructions across the tree of life (Laumer et al., 2019; Pizarro et al., 2018; Wickett et al., 2014), and scleractinian corals are no exception. Molecular phylogenies for Scleractinia were first reconstructed in the mid‐1990s, based on single‐gene comparisons of either nuclear or mitochondrial ribosomal genes (Chen, Odorico, Tenlohuis, Veron, & Miller, 1995; Romano & Palumbi, 1996, 1997). Since then, multigene analyses with mitochondrial and nuclear markers have been performed (Arrigoni, Berumen, et al., 2014; Arrigoni, Terraneo, et al., 2014; Fukami et al., 2004, 2008; Huang et al., 2016; Huang, Benzoni, Arrigoni, et al., 2014; Huang, Benzoni, Fukami, et al., 2014). Data sets with up to 12 loci are now available, but these have patchy gene coverage among species (Hartmann, Baird, Knowlton, & Huang, 2017; Huang, 2012; Huang & Roy, 2015; Kitahara et al., 2016). Phylogenetic matrices with such limited number of genes are prone to producing uncertain branch length or age estimates and even erroneous species tree topologies (Rokas, Williams, King, & Carroll, 2003; Zhu, Dos Reis, & Yang, 2015).

Today, high‐throughput, next‐generation sequencing (NGS) platforms have enabled vast amounts of data to be harnessed at relatively low cost (Goodwin, McPherson, & McCombie, 2016; Kulkarni & Frommolt, 2017). Whole genome data, while ideal, remain a distant possibility for Anthozoa due to challenges in whole genome assembly (Sohn & Nam, 2018), especially because of diverse microbial symbionts contaminating the sequencing reads (Artamonova & Mushegian, 2013). Indeed, recent anthozoan whole genome analyses comprise fewer than 15 taxa (Cunning, Bay, Gillette, Baker, & Traylor‐Knowles, 2018; Ying et al., 2018). Therefore, alternative NGS methods tailored for phylogenomics are applied more widely among Anthozoa, and include restriction site‐associated DNA sequencing (RADseq) and genome skimming for shallow relationships (Forsman et al., 2017; Johnston et al., 2017), as well as phylotranscriptomics (Lin et al., 2016; Quek & Huang, 2019; Richards, Carvajal, Wallace, & Wilson, 2019; Zapata et al., 2015) and target enrichment via hybrid capture (Quattrini et al., 2018) for more inclusive taxon sets.

Capitalizing on recent technological advancements, the hybrid capture approach has been steadily gaining traction over the last decade (Bossert & Danforth, 2018; Bragg, Potter, Bi, & Moritz, 2016; Faircloth et al., 2012; Lemmon, Emme, & Lemmon, 2012). Principally, DNA or RNA probes are designed based on conserved sequences within the genome and used to hybridize to these targeted loci in a DNA library that are subsequently enriched and sequenced. This method has the potential to target hundreds to thousands of homologous loci simultaneously in a cost‐effective fashion by pooling a number of samples together in a single hybridization reaction (e.g., *n* = 96; Liu et al., 2019). Target enrichment has been applied successfully in several marine taxa, including percomorphs (Dornburg et al., 2017), ophiuroids (Hugall, O’Hara, Hunjan, Nilsen, & Moussalli, 2016), mobulids (White et al., 2018) and anthozoans (Quattrini et al., 2018). In particular, the baits designed by Quattrini et al. (2018) were based on both ultraconserved elements and transcriptomes, and were tested in vitro on 33 anthozoan taxa, including just four scleractinians. A further nine genome‐enabled taxa were included for phylogenetic analyses which included two scleractinians.

In this study, we designed, screened and tested target‐enrichment baits based on 44 scleractinian transcriptomes for the broad purpose of phylogenetic reconstruction among stony corals. Baits designed in this study were tested on 18 species across both the “Robust” and “Complex” clades (sensu Romano & Palumbi, 1996), as well as two corallimorpharian outgroups. With judicious selection of putatively scleractinian loci post‐capture, we demonstrate that our baits are able to capture orthologous markers across the scleractinian tree for large‐scale phylogenomic analysis.

## 2 MATERIALS AND METHODS

### 2.1 Bait design and screening

Scleractinian transcriptomes for 43 terminals from 39 species analyzed in Quek and Huang (2019) were translated into their amino acid sequences in Qiagen CLC Genomics Workbench v9.5.4. Clustering of orthologs was performed in orthofinder v1.1.8 (Emms & Kelly, 2015) under default settings with the *diamond_more_sensitive* flag activated (Buchfink, Xie, & Huson, 2015). Single‐copy orthologs were extracted for bait design.

To ensure an even phylogenetic representation for each locus, all single‐copy orthologs selected must be represented by a minimum of six (out of 39) scleractinian taxa, of which two must belong to either the “Robust” or “Complex” clade (sensu Romano & Palumbi, 1996). A number of filtering steps were incorporated to retain only putatively scleractinian loci. A blastp (*e*‐value = 10^–6^) of all identified sequences was conducted against the gene models of 10 publicly available coral genomes (Table 1). Only orthologs with at least one transcript that had a positive hit to a gene model with bit‐score ≥50 were kept. To filter out potential non‐scleractinian transcripts originating from the coral holobiont, we conducted a local blastn (*e*‐value = 10^–6^) of aforementioned positive hits against GenBank data (downloaded October 2018). A single best hit for each transcript was identified via sorting blastn hits by highest bit‐score, lowest e‐value and highest percentage identity. Transcripts matched to a non‐cnidarian with ≥80% sequence similarity across an alignment length ≥100 bp were removed. If the removal of transcript(s) resulted in an ortholog being represented by less than six taxa, the ortholog was removed altogether.

**TABLE 1:** Reference genomes used for putatively scleractinian transcript identification and bait design

Species	Access	Reference
**Scleractinia**		
*Acropora digitifera*	http://marinegenomics.oist.jp/	Shinzato et al. (2011)
*Acropora tenuis*	http://refuge2020.reefgenomics.org/	ReFuGe 2020 Consortium (2015)^a^
*Fungia* sp.	http://refuge2020.reefgenomics.org/	Ying et al. (2018)^a^
*Galaxea fascicularis*	http://refuge2020.reefgenomics.org/	Ying et al. (2018)^a^
*Coelastrea aspera*	http://refuge2020.reefgenomics.org/	Ying et al. (2018)^a^
*Montastraea cavernosa*	http://matzlab.weebly.com/data‐‐code.html	
*Orbicella faveolata*	GCF_002042975.1	
*Pocillopora damicornis*	http://pdam.reefgenomics.org/	Cunning et al. (2018)^a^
*Porites lutea*	http://refuge2020.reefgenomics.org/	Ying et al. (2018)^a^
*Stylophora pistillata*	http://spis.reefgenomics.org/	Voolstra et al. (2017)^a^
**Symbiodiniaceae**		
*Breviolum minutum*	http//marinegenomics.oist.jp/	Shoguchi et al. (2013)
*Cladocopium goreaui*	http://symbs.reefgenomics.org/	Liu et al. (2018)^a^
*Cladocopium* sp.	http://marinegenomics.oist.jp/	Shoguchi et al. (2018)
*Fugacium kawagutii*	http://symbs.reefgenomics.org/	Liu et al. (2018)^a^
*Symbiodinium microadriaticum*	http://smic.reefgenomics.org/	Aranda et al. (2016)^a^
*Symbiodinium* sp.	https://marinegenomics.oist.jp/	Shoguchi et al. (2018)

^a^ Reefgenomics.org: Liew, Aranda, and Voolstra (2016).

For the remaining transcripts, we conducted another blastp (*e*‐value = 10^–6^) against the same gene models as above. Positive hits were then sorted by highest bit‐score, lowest e‐value and highest percentage identity, extracting the best hit for each ortholog that was then placed in one of 10 bins, each representing one of the 10 reference genomes (Table 1). Orthologous amino acid sequences were first aligned using mafft v7.3.10 with the L‐INS‐i method (Katoh & Standley, 2013), and then back‐translated using pal2nal v14 (Suyama, Torrents, & Bork, 2006) into their corresponding nucleotide sequences.

Baits were designed using baitfisher v1.2.8 (Mayer et al., 2016) with alignment cutting performed separately for each bin based on the respective reference genome. To maximize taxon and loci recoverability (Schott et al., 2017), we designed multiple 120 bp baits across different lengths of loci located by baitfisher in the genome following Bank et al. (2017). We first required that seven baits were designed across a 240 bp window with a 20 bp offset between each bait. Shorter windows were used iteratively following failure to locate a suitable region after alignment cutting: five tiled baits in a 200 bp window; three tiled baits in a 160 bp window; and finally, one bait for a 120 bp window.

To generate a single bait set that contained the best bait locus following alignment cutting, we used baitfilter v1.0.6 (Mayer et al., 2016) (*‐m fs*) for each of the 10 bait sets designed. As a final check for baits possibly binding to noncoral DNA, we mapped the extracted baits at 70% sequence similarity and 70% length using CLC Genomics Workbench v9.5.4 and searched by blastn (*e*‐value = 10^–4^) against six Symbiodiniaceae genomes (Table 1). All mapped or blastn‐matched baits were removed. If there were ≥3 baits removed within a bait region, or only one remaining bait, we removed the entire bait region. Any duplicated baits were removed using seqkit v0.7.2 *rmdup* package (Shen, Le, Li, & Hu, 2016). Finally, we removed baits that had the potential to self‐hybridize by running a blastn of the baits against one another, searching for baits with regions that were reverse complementary to other baits.

To help flag cross‐contamination post‐capture and potential sample misidentification, we designed barcoding baits targeting mitochondrial cytochrome c oxidase subunit I (COI) and nuclear histone H3. These genes are suitable for identification to genus (Arrigoni, Berumen, et al., 2014; Huang, Benzoni, Arrigoni, et al., 2014; Huang, Meier, Todd, & Chou, 2008) and can be used to confirm the identities of potentially misidentified or erroneously labelled samples for a broadscale phylogeny. We first downloaded 129 COI and 32 histone H3 sequences from GenBank and obtained 16 additional histone H3 sequences from samples collected in Singapore via Sanger sequencing following Huang, Licuanan, Baird, and Fukami (2011) (Table S1). Taxon coverage spanned the scleractinian phylogeny. baitfisher v1.2.7 (Mayer et al., 2016) was used to design 120 bp baits with an offset of 20 bp across the entire length of each gene. Optimal baits were identified using baitfilter v1.0.6 (*‐m as*) and added to the final bait set, labelled as either “coi” or “h3” and can be removed at the user's discretion.

The full set of biotinylated RNA probes were manufactured by Arbor Biosciences (myBaits Custom Target Capture Kit, USA).

### 2.2 Sample collection, target enrichment and sequencing

A total of 18 coral samples, each belonging to a genus analyzed in Quek and Huang (2019), as well as two corallimorpharians, were collected from Singapore reefs (Table S2). Fragments of corals were stored in either 100% ethanol or RNAlater (Invitrogen) until DNA extraction. The remaining skeletal vouchers were treated with a powerful waterjet to remove coral tissue, bleached in dilute sodium hypochlorite, then washed and dried. Voucher specimens were deposited at the Lee Kong Chian Natural History Museum (Table S2).

High quality gDNA was extracted following a modified protocol suggested by Forsman et al. (2017). Briefly, coral samples were crushed and DNA extraction was carried out using E.Z.N.A Mollusc DNA Kit (Omega Bio‐tek) with a modified two‐elution step. For the first elution, only 35 ul of elution buffer (0.10 nM Tris‐HCl) was added to the column, removing a majority of small molecular‐weight, fragmented DNA. In the second step, 50 μl of elution buffer was added four times, resulting in a total gDNA volume of 200 μl. High quality, eluted gDNA (200 μl) from the E.Z.N.A. Mollusc DNA Kit extraction was then purified using Zymo Genomic DNA Clean and Concentrator. Four rounds of DNA elution were conducted post‐cleanup, with 15 ul of elution buffer added per round to a final volume of 60 μl.

Libraries were prepared by first sonicating purified gDNA using Bioruptor Pico (Diagenode) with a target mode size of 200 bp. Adapters were ligated with KAPA dual‐indexed adapters for Illumina platforms (KK8722; KAPA Biosystems) using the KAPA HyperPrep Kit (KK8502; KAPA Biosystems), according to manufacturer's recommendations. An additional double‐sided size selection was carried out in the final step to narrow fragment size distribution in final libraries according to KAPA Biosystems protocol using Agencourt AMPure XP beads (Beckman Coulter). Libraries were eluted in 20 μl of Tris‐HCl buffer and 2 μl was used for quantification using a Qubit 3 Fluorometer. Five libraries were pooled randomly in equimolar ratios (~100 ng per sample) to a total of 500 ng of DNA per pool, and concentrated to 7 μl per pool (71.42 ng/μl) for hybrid capture.

Hybrid capture was executed following the manufacturer's protocol (myBaits Custom Target Capture Kit; Arbor Biosciences) with 14 cycles of post‐capture amplification. Amplified libraries were purified using Agencourt AMPure XP beads (Beckman Coulter) and eluted in 20 μl of nuclease‐free water. DNA concentration was quantified using a Qubit 3 Fluorometer and the 20 libraries were pooled in equimolar concentrations. Libraries were sequenced on a single lane of Illumina HiSeq 4000 (150 × 150 bp).

### 2.3 Sequence assembly and quality filtering

Raw reads were demultiplexed and processed by trimming low quality bases and adapters using trimmomatic v0.38 (Bolger, Lohse, & Usadel, 2014) under default settings. In order to identify and assemble orthologous loci targeted by the baits, processed paired reads were parsed into hybpiper v1.2 (Johnson et al., 2016) to locate targeted exons. Reads were first mapped to the transcripts that were used for bait design (henceforth referred as ‘baitfile’) using bwa v0.7.17 (Li, 2013) under default hybpiper settings. Mapped reads were assembled into contigs with spades v3.12.0 (Bankevich et al., 2012) and exonic regions were identified using exonerate v2.2.0 (http://github.com/nathanweeks/exonerate).

Verification of cross‐contamination was first conducted by running the following barcoding check. Trimmed reads were first mapped to COI and histone H3 sequences from 12 samples—six “Robust” and five “Complex” corals spanning eight families and one corallimorpharian *Rhodactis indosinensis* (Table 2)—using bwa mem under default settings (Li, 2013). Mapped reads were extracted in FASTQ format using samtools (Li et al., 2009) and assembled using spades v3.12.0 under default settings with the *‐‐careful* flag activated (Bankevich et al., 2012). Assembled contigs with a minimum length of 200 bp were searched by blastn against the assembled Sanger sequences. Positive hits were then filtered for the single best hit with the highest sequence similarity (≥98%) over ≥200 bp to check sample identity.

**TABLE 2:** Summary statistics of loci assembled per sample for both exons‐only and exons + supercontigs data sets

Species	Number of loci (#/%)	Locus length range (bp) (exons‐only/supercontigs + exons)	Mean locus length (±*SD* bp) (exons‐only/supercontigs + exons)
**Robust corals**			
*Cyphastrea serailia* ^a^	386/85.40	93–1,923/156–4,356	443 ± 223/847 ± 597
*Diploastrea heliopora*	401/88.71	87–1,920/117–4,414	444 ± 228/802 ± 555
*Dipsastraea maxima* ^a^	381/84.29	93–1,611/126–4,694	432 ± 194/853 ± 594
*Goniastrea retiformis*	393/86.95	93–1,395/99–4,479	439 ± 199/860 ± 608
*Herpolitha limax* ^a^	349/77.21	93–1,209/126–3,690	425 ± 194/834 ± 560
*Lobophyllia radians*	389/86.06	93–1,572/117–3,619	435 ± 203/794 ± 520
*Oulastrea crispata* ^a^	323/71.46	81–1,878/135–4,247	425 ± 236/798 ± 569
*Platygyra sinensis* ^a^	383/84.73	90–1,674/192–3,950	443 ± 215/834 ± 583
*Plesiastrea versipora* ^a^	354/78.32	93–1,383/147–5,192	437 ± 209/823 ± 564
*Pocillopora acuta*	258/57.08	123–1,587/153–3,531	473 ± 234/833 ± 523
**Complex corals**			
*Acropora aspera*	263/58.19	111–1,938/144–5,286	490 ± 238/1,005 ± 714
*Astreopora expansa* ^a^	219/48.45	72–1,413/189–2,818	424 ± 209/774 ± 507
*Fimbriaphyllia ancora* ^a^	343/75.88	90–2,895/105–6,837	513 ± 322/998 ± 765
*Galaxea astreata* ^a^	343/75.88	99–2,697/105–8,843	521 ± 310/1,053 ± 871
*Goniopora lobata*	277/61.28	54–1,521/114–5,123	430 ± 214/693 ± 498
*Pachyseris speciosa* ^a^	325/71.90	66–2,889/147–4,947	475 ± 288/899 ± 659
*Porites lobata* ^a^	311/68.81	54–2,208/63–6,595	461 ± 259/875 ± 676
*Turbinaria mesenterina*	329/72.79	96–3,798/111–5,537	463 ± 312/934 ± 693
**Corallimorpharia**			
*Rhodactis inchoata*	43/9.51	183–717/183–1,773	375 ± 144/541 ± 352
*Rhodactis indosinensis* ^a^	47/10.40	138–759/138–1,291	356 ± 123/448 ± 226

Note

Percentage of loci is based on total number of loci (*n* = 452). A supercontig includes both exon and intron regions in a sequence.

^a^ Samples used in identity checks with COI and histone H3 barcodes.

Having verified that there were low levels of cross‐contamination between samples (see 3.1 Bait design and screening), we reconstructed gene trees for loci located by hybpiper using fasttree v2.1.9 (Price, Dehal, & Arkin, 2010). Gene trees were based on coding sequences and separated into two sets, either potentially paralogous loci or single‐copy loci as identified by hybpiper. The visualization of gene trees served several functions: (a) to remove potentially paralogous sequences; (b) to check for contamination, and (c) to check for locus capture efficiency. We treated paralogous loci following Johnson et al. (2016) and kept the “.main” paralog; or the “0.0” paralog if no “.main” was present for Type I paralogs (recent duplicates or alleles). For genes indicative of type II paralogy (deep divergences or early gene/genome duplications), we conservatively removed the loci from further downstream analyses.

Contamination was checked first by visually inspecting gene trees and using the following criteria to remove contaminant sequences: (a) if a paralogous locus had two sequences with one in the expected major clade and the other not (e.g., a “Complex” coral with a paralogous locus having one sequence in the “Complex” clade and the other in the “Robust” clade), the contaminant sequence was removed; (b) if a taxon for a single‐copy locus was placed in the wrong major clade (e.g., “Robust” coral in the “Complex” clade), the taxon was removed, and (c) if a taxon/clade exhibited an unusually long branch within a tree, the taxon/clade was removed from the locus. Finally, based on the gene trees, we removed loci which captured less than three scleractinian taxa, gave spurious topologies, or were phylogenetically uninformative (i.e., poor or no phylogenetic signal due to highly conserved gene sequences).

For all loci and barcodes retained, read coverage was determined with the following pipeline. Trimmed reads were first mapped to assembled contigs (*n* = 18 samples) and barcode(s) (*n* = 12 samples for COI; *n* = 8 samples for histone H3) using bwa mem under default settings (Li, 2013). Mapped reads were extracted using samtools in BAM format (Li et al., 2009) and funneled into qualimap v2.2.1 (Okonechnikov, Conesa & García‐Alcalde, 2016) to compute the mean coverage per locus.

Finally, to determine if mitochondrial contigs could be recovered at mitochondrial loci other than COI, thereby allowing for the safe removal of barcoding baits, we conducted a similar analysis to that of Quek, Chang, Ip, and Huang (2019). Briefly, trimmed reads for all scleractinian samples (*n* = 18) were assembled by spades v3.1.2 under default settings. Mitochondrial contigs assembled were then identified by executing a blastn of all contigs to either the mitogenome or mitochondrial genes of a closely‐related taxon; all contigs with sequence similarity of 90% with an overlap of 200 bp were extracted. Contigs were then assembled using cap3 (Huang & Madan, 1999) and annotated using mitos2 (Bernt et al., 2013).

### 2.4 Phylogenetic inference

Two separate data matrices were prepared for phylogenetic analysis. In the first data set, we combined only filtered coding sequences for both paralogy‐filtered loci and single‐copy loci (exons‐only data set). In the second, we included filtered sequences with both introns and exons (supercontigs in hybpiper) for nonparalogous sequences and combined it with the paralogy‐filtered coding sequences (exons + supercontigs data set). Sequences were first aligned for each locus in mafft v7.427 with the L‐INS‐i method (Katoh & Standley, 2013), and poorly aligned regions were trimmed using trimal v1.4 under the heuristic setting (Capella‐Gutiérrez, Silla‐Martínez, & Gabaldón, 2009). Trimmed alignments were concatenated into a single matrix and partitioned by loci (*n* = 452) for the respective data sets.

For each data set, the maximum likelihood (ML) phylogeny was reconstructed using raxml v8.2.11 (Stamatakis, 2014) under the rapid hill climbing mode and GTRGAMMA substitution model (100 random tree searches and 500 bootstrap pseudoreplicates). Bayesian analysis was performed using exabayes v1.5 (Aberer, Kobert, & Stamatakis, 2014), generating four coupled Markov chain Monte Carlo chains in four independent runs, each with 3 million generations and sampling every 500 generations. Convergence was checked based on average standard deviation of split frequencies (ASDSF < 0.001%). A consensus tree across all four runs was generated after the first 25% of generations had been discarded as burnin.

**FIGURE 1:**
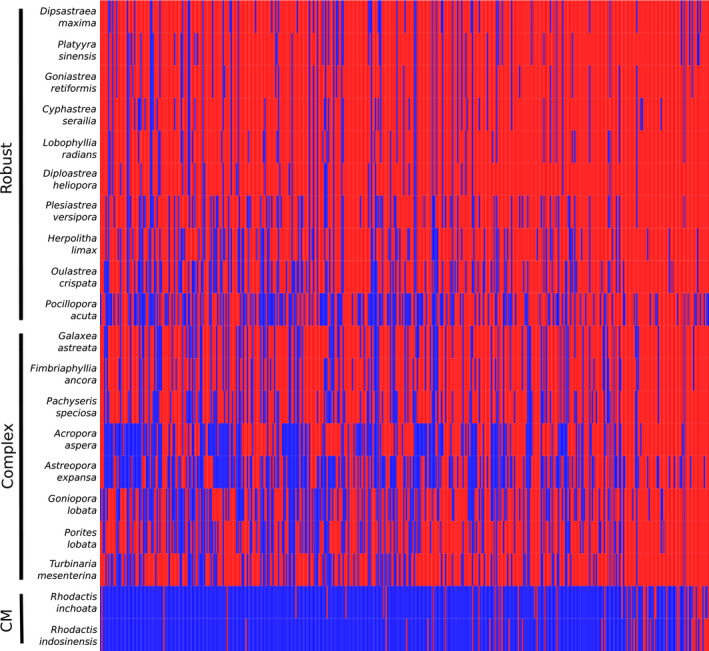
Coverage of loci captured by target‐enrichment baits as determined by hybpiper post‐filtering (blue = absent, red = present)

For the exons‐only data set, we further reconstructed a ML phylogeny for each loci with at least four taxa (*n* = 438) using raxml v8.2.11 (Stamatakis, 2014) under the rapid hill climbing mode and GTRGAMMA substitution model (10 random tree searches and 100 bootstrap pseudoreplicates). The gene trees were input for species tree analysis using astral‐iii v5.6.3 (Zhang, Rabiee, Sayyari, & Mirarab, 2018). Low support branches (<10% bootstrap support) were removed from all gene trees using newick utilities (Junier & Zdobnov, 2010) as per developers’ recommendation. Finally, gene tree incongruence relative to the species tree (Figure 1) was assessed with discovista (Sayyari, Whitfield, & Mirarab, 2018) based on family‐level splits (bootstrap support ≥75 as recommended).

## 3 RESULTS

### 3.1 Bait design and screening

From an initial 3,567 potential loci identified by orthofinder v1.1.8 (Emms & Kelly, 2015), our blastp returned 853 positive hits (bit‐score ≥50). Following the blastn filter, we retained a total of 842 alignments for bait design. With alignment to genomes and bait tiling, a total of 665 loci had baits designed over 1,267 exon regions. After the final mapping to Symbiodiniaceae, removal of self‐hybridizing baits and duplicates, we retained a total of 15,919 baits for 605 orthogroups over 1,139 exon regions. A further 236 and 62 baits were designed for COI and histone H3 genes respectively. The reference genomes used with the respective number of alignments, features and baits designed are detailed in Table S3.

All raw sequencing reads are available as NCBI Sequence Read Archive under BioProject accession number PRJNA602211. Following demultiplexing, the total number of reads per sample was between 6,220,293 and 16,119,962 (mean 10,027,194 ± *SD* 2,511,802; Table S4). The total number of trimmed reads remaining that were mapped to the baitfile in hybpiper was between 4,941,626 and 14,368,535 per sample (mean 8,617,176 ± *SD* 2,216,674; Table S4). The proportion of reads that mapped to sequenced barcodes ranged from 18.45% to 51.06% (mean 32.30% ± *SD* 10.44%; Table S4). In accordance with expectations of cross‐sample contamination (Bank et al., 2017; Hugall et al., 2016), the assembly of barcoding baits revealed that a sample may have several sequences passing the filtering criteria with the best blastn hit to a nontarget taxon. However, these matches could be recognized and disregarded as the k‐mer coverage was at least 100 times lower than that of the correct hit, so we could recover the correct samples based on one or both barcoding genes. In total, we recovered accurate COI barcodes for all 11 tested scleractinians and a corallimorpharian, as well as histone H3 barcodes for eight scleractinians and a corallimorpharian. A summary of the barcoding results is available at Zenodo (http://dx.doi.org/10.5281/zenodo.3590246; barcoding_baits_summary.csv).

Read coverage was high across all loci captured, ranging from a mean coverage read depth of 749 (±*SD* 4,826) in *Goniopora lobata* to 3,424 (±*SD* 3,223) in *Acropora aspera*, with an overall mean coverage of 2,201 (±*SD* 676) across all samples (*n* = 18). This coverage was ~150× lower than that of the barcoding baits, with a mean read depth of 366,183 (±*SD* 194,946) for COI (*n* = 11) and 323,547 (±*SD* 261,479) for histone H3 (*n* = 8). We were able to recover a majority of mitochondrial genes from each sample (Figure S2), with the exception of species without suitably close relatives represented among GenBank's pool of mitogenomes (*Diploastrea heliopora, Herpolitha limax*, *Lobophyllia radians* and *Oulastrea crispata*).

Following stringent quality filtering of loci to remove paralogs, contaminant sequences and uninformative loci, we obtained a final bait set targeting 452 putatively scleractinian loci (mean 531 ± *SD* 307 bp) over 865 exon regions. The length of contigs recovered ranged from 54 bp for *Goniopora lobata* and *Porites lobata,* to 3,798 bp for *Turbinaria mesenterina* based on the exons‐only data set (mean 453 ± *SD* 242 bp); and from 63 bp for *Porites lobata* to 8,843 bp for *Galaxea astreata* based on the exons + supercontigs data set (mean 857 ± *SD* 625 bp) (Table 2). The original (15,919 baits) and filtered (13,479 baits) bait sets, as well as baitfile comprising the final 452 loci targeted are available at Zenodo (http://dx.doi.org/10.5281/zenodo.3590246; baits_designed.fa, baits_designed_filtered.fa and baitfile_452.fa respectively).

### 3.2 Phylogenetic inference

Of the 605 loci targeted, hybpiper was able to generate contigs for 581 loci. Following filtering of loci to remove paralogs (43 loci), contaminant sequences (154 sequences) and uninformative loci (129 loci, including those with <3 taxa captured), a total of 452 loci spanning 865 exon regions were retained for phylogenomic inference. Loci recovered across scleractinian samples were fairly evenly distributed, with completeness ranging from 48.45% to 88.71% (mean 74.08% ± *SD* 11.65%; Table 2; Figure 1). The average taxon occupancy of the final matrix was 13.33 (±*SD* 4.14) scleractinians per locus, out of 18 scleractinians tested. Contigs assembled for each locus and concatenated alignments are available at Zenodo (http://dx.doi.org/10.5281/zenodo.3590246). Post‐trimming, the concatenated matrices contained a total of 201,137 sites with 30.86% missing data (following Quek & Huang, 2019) for the exons‐only data set (69.14% complete; mean alignment length = 456 ± *SD* 233 bp), and 287,749 sites with 32.43% missing data for the exons + supercontigs data set (67.57% complete; mean alignment length = 636 ± *SD* 352 bp). The latter showed greater sequence variability with 41.39% parsimony‐informative sites, compared to 34.30% in the former data set (Table 3).

**TABLE 3:** Concatenated matrix statistics for both exons‐only and exons + supercontigs data sets

Data set	Missing data (%)	Concatenated matrix length (bp)	Mean locus length (±*SD* bp)	Locus length range (bp)	Parsimony informative sites (#/%)
Exons‐only	30.86	201,137	456 ± 233	6–2133	68,997/34.30
Exons + supercontigs	32.43	287,749	636 ± 351	54–2491	119,095/41.39

Note

Missing data percentages as defined in Quek and Huang (2019).

A supercontig includes both exon and intron regions in a sequence.

**FIGURE 2:**
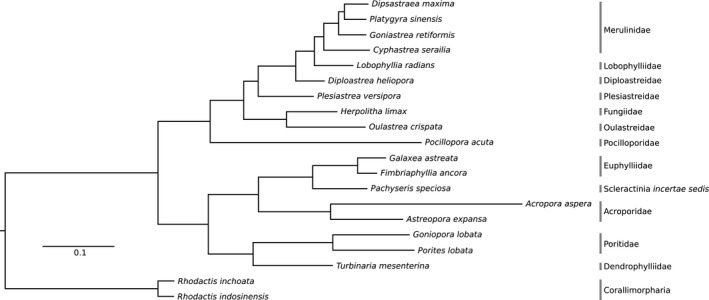
Maximum likelihood phylogeny of Scleractinia for exons‐only data set (minimum taxon occupancy of three scleractinian taxa per locus; 30.86% missing data; 452 loci over 865 exon regions; 201,137 bp) with *Rhodactis* as outgroup. All nodes have maximum bootstrap values and posterior probabilities

All phylogenetic trees inferred—from maximum likelihood, Bayesian and species tree analyses—were congruent with maximum bootstrap values and posterior probabilities at all nodes (Figure 2 and S3; available at Zenodo, http://dx.doi.org/10.5281/zenodo.3590246). The “Robust” and “Complex” clades, as well as monophyletic families Acroporidae, Euphylliidae, Poritidae and Merulinidae were unambiguously recovered. Furthermore, the vast majority of gene trees analyzed supported the phylogeny in both the concatenated and species tree reconstructions (Figure S1).

## 4 DISCUSSION

In this study, we have designed hybrid‐capture baits to target 605 putatively scleractinian loci over 1,139 exon regions. Laboratory testing of the baits shows that they are highly accurate and specific, enriching 452 loci across 865 exons that map to coral genomes following rigorous post‐sequencing filtering. Our test on 18 species spanning 18 genera and 12 families also demonstrate that our baits are able to capture loci effectively—despite using just a minimum of six scleractinian taxa per locus for bait design—with minimal taxonomic bias across the “Robust” and “Complex” clades (Figure 1), which account for >98% of all species in Scleractinia (Huang, 2012; Huang & Roy, 2015; Kitahara et al., 2016). A slight bias towards the capture of “Robust” (mean 80% ± *SD* 9.7%) relative to “Complex” corals (mean 67% ± 9.8%) might be due to the larger number of baits designed based on “Robust” coral genomes (*n* = 9,149) compared to “Complex” coral genomes (*n* = 6,770). Future studies need to verify the efficacy of these baits in recovering sequences from the “Basal” clade (Stolarski et al., 2011). Nevertheless, our analyses have recovered a maximally‐supported phylogeny, with a topology congruent with recent broad‐scale phylogenies (Figure 2; Kitahara et al., 2016; Quek & Huang, 2019). We noted a slightly elevated level of gene tree incongruence among members of the “Robust” clade (Figure S1), which could be attributed to factors such as incomplete lineage sorting (Woodhams, Lockhart, & Holland, 2016) and varying phylogenetic signal between clades (Gonçalves, Simpson, Ortiz, Shimizu, & Jansen, 2019). Analyses similar to that of Ying et al. (2018) performed at a broader scale would reveal factors driving these differences.

The method we employed in designing target‐enrichment baits involves multiple filtering steps to capture putatively scleractinian loci. Considering the diversity of the coral holobiont (Stat et al., 2012; Thompson, Rivera, Closek, & Medina, 2014; Wainwright, Afiq‐Rosli, Zahn, & Huang, 2019), a number of transcripts assembled would inevitably be of noncoral origins despite preliminary filters for Symbiodiniaceae transcripts (Quek & Huang, 2019). We circumvented symbiont contamination by leveraging a number of published coral genomes, using both blastp to locate putatively coral loci and alignment cutting used for bait design. In other words, our protocol ensured that the targeted loci were present in at least one of the reference genomes. Genome‐based bait design is useful for locating intron‐exon boundaries for optimal bait design (Bank et al., 2017; Hugall et al., 2016), and specifically in this study, the approach further aids in the accurate identification of coral loci.

In our post‐sequencing analysis, we took advantage of a unique phylogenetic signature of Scleractinia: the deep split between the “Robust” and “Complex” clades (Huang, 2012; Huang & Roy, 2015; Kitahara, Cairns, Stolarski, Blair, & Miller, 2010; Kitahara et al., 2016; Romano & Palumbi, 1996, 1997; Stolarski et al., 2011; Ying et al., 2018). By inspecting individual gene trees for this pattern, we ensured that contaminant sequences were appropriately removed, and only phylogenetically informative, putatively coral and orthologous loci were retained for phylogenomic reconstruction. While laborious for a large number of loci, we recommend using well‐substantiated prior information to help select loci for future target enrichment studies. We note that the identification of paralogs can now be expedited by newly‐developed tools such as Clan_Check (Siu‐Ting et al., 2019), which detects potential instances of hidden paralogy from a large number of trees, highlighting genes that may warrant further investigation (e.g., visual inspection of gene trees and sequence alignments, etc). Ultimately, meticulous data curation is of utmost importance in any phylogenetic reconstruction; molecular data sets are susceptible to cross‐contamination (Bank et al., 2017; Hugall et al., 2016) and paralogy (Siu‐Ting et al., 2019), both of which compound inaccuracies in phylogenomic analyses and therefore ought to be carefully checked.

While only exon sequences have been used for all phylogenomic analyses due to the divergent taxa sampled, we also provide an alternative pipeline to include intron sequences. Contigs comprising both exon and intron regions (supercontigs) can readily be extracted from hybpiper (Johnson et al., 2016). Since introns are more variable than exons (Table 3; Thomson, Wang, & Johnson, 2010), they may be useful for clarifying cryptic species complexes and resolving shallow divergences (Concepcion, Crepeau, Wagner, Kahng, & Toonen, 2008; Oppen, Willis, Vugt, & Miller, 2000; Pinzón & LaJeunesse, 2011).

Beyond the recovery of phylogenetic relationships, a handy aspect of the bait set designed here lies in the inclusion of baits targeting barcodes that are highly enriched in our assemblies. Considering that coral taxa are notoriously difficult to tell apart and taxonomic misidentifications even among families are not uncommon (e.g., *Turbinaria* sp. identified as *Astreopora* sp., noted in Quek & Huang, 2019), we provide an additional safeguard in the form of baits designed to assign samples to their genera. Furthermore, preliminary quality checks estimating the levels of cross‐contamination can be assessed based on the number of contigs and depth of sequencing recovered per barcode. Nearly one‐third of our reads mapped to both barcoding baits, which is high when compared to previous target‐enrichment studies (e.g., 5% of all reads originating from COI in Hugall et al., 2016). However, the two barcoding loci we used are essential for reliable verification of sample identities, and confer redundancy in case a single locus is not recovered for any one sample. Indeed, nine samples in Hugall et al. (2016) did not retain COI barcodes, while one and three of our samples did not recover COI and histone H3, respectively, but every sample had at least one barcoding locus. Ultimately, we have been able to obtain numerous loci per sample that contribute to robust, consistent inferences despite the large proportion of barcode reads produced.

As mitochondrial DNA is naturally enriched, sequencing reads of target‐enriched libraries typically include background mitochondrial sequences as byproducts. For example, Allio et al. (2019) were able to extract COI barcodes and other mitochondrial genes from 501 hybrid‐capture libraries in ants (Formicidae), and Taucce et al. (2018) assembled mitogenomes for five frog species (Anura) from hybrid‐capture libraries. Here, we show that mitochondrial genes other than COI could be recovered for all samples in this study (Figure S2). Despite having on average >2,000× read depth across all 452 loci targeted, both COI and histone H3 have higher read depth (~150×) in comparison. The lower number of reads for the targeted loci could raise sequencing cost, particularly with lower throughput sequencers (e.g., Illumina MiSeq), but clearly, without targeted capture of these barcodes, mitochondrial loci may not be consistently captured even if they are naturally enriched (Figure S2).

We stress that the barcodes have been designed as a safeguard against sample misidentification, and it is at researchers’ discretion to remove these barcodes. In particular, their removal is recommended if: (a) there is little to no risk of sample misidentification or mix‐ups; (b) a low‐throughput sequencing strategy is employed, or (c) a lower read depth is an acceptable tradeoff to maximize the number of samples sequenced. Furthermore, the depth of sequencing reflected in this study suggests that a few hundred samples can be safely combined into a single sequencing run with still sufficient read depth, especially with the removal of barcoding baits. Finally, it must be emphasized that these baits are useful for identification up to genus level. Where necessary, we strongly recommend the design of more specific baits, such as *Pax‐C* 46/47 intron for *Acropora* spp. (Márquez, Van Oppen, Willis, Reyes & Miller, 2002; Van Oppen, Willis, Van Vugt & Miller, 2003) and open reading frame region for *Pocillopora* spp. (Flot & Tillier, 2006; Schmidt‐Roach, Miller, Lundgren & Andreakis, 2014) following the pipeline outlined above.

The target‐enrichment baits and sequence processing method presented here—leveraging recent developments in molecular techniques, sequencing and bioinformatics—represent another major step towards building large, gene‐rich scleractinian trees. In particular, Quattrini et al. (2018) had designed baits targeting the more inclusive clade of Anthozoa. The baits designed were tested in vitro on 33 anthozoan taxa (four scleractinians), with a further nine genome‐enabled taxa (two scleractinians) included for phylogenetic reconstruction. The data set thus incorporated a total of 42 anthozoans comprising 22 hexacorals (six scleractinians) and 20 octocorals. When comparing our phylogenetic data matrix (452 loci of the exons‐only data set) with theirs containing a similar number of loci (438 loci of the 50% taxon occupancy matrix for Hexacorallia), there are 68,997 parsimony‐informative sites in the former, more than twice the 34,390 parsimony‐informative sites in the latter. Only when the total amount of missing data in Quattrini et al. (2018) are increased by lowering the taxon occupancy to 25% does the number of parsimony‐informative sites increase to 63,968. The bait set designed in our study is clearly highly specific and targeted towards scleractinians, with much lower coverage for the sister‐group corallimorpharians (Figure 1). Not surprisingly then, a blastn of the baits in Quattrini et al. (2018) against our final set of 13,479 baits reveals no overlap between the loci targeted, highlighting immense bait dissimilarities as a result of the distinct taxonomic level targeted (Shaffer, McCartney‐Melstad, Near, Mount, & Spinks, 2017). Taken together, we suggest combining the two bait sets in future studies of Anthozoa to maximize the loci captured for scleractinian corals.

Resolving the phylogeny of scleractinian corals is critical for elucidating processes related to their evolutionary success and trajectories based on comparative genomics (Bhattacharya et al., 2016; Ying et al., 2018), and for reconstructing their origin and trait evolution (Hartmann et al., 2017; Madin et al., 2016; Stolarski et al., 2011). To date, the largest coral phylogeny reconstructed using NGS data includes only 39 scleractinian species represented by 43 samples (Quek & Huang, 2019). Over the next few years, we aim to increase the number of taxa placed on the phylogenomic tree by several fold using the method developed here to advance our understanding of coral evolution.

## AUTHOR CONTRIBUTIONS

R. Z. B. Q., G. W. R., and D. H. conceptualized the study. R. Z. B. Q., S. S. J., and M. L. N. collected the samples. R. Z. B. Q. conducted the laboratory work, designed the pipeline, and analyzed the data. The manuscript was written by R. Z. B. Q., and D. H., with input from all authors. Publication of this manuscript was approved by all authors.

## Supporting information

Supplementary Material

## Data Availability

Baits designed, bait file, contigs assembled and scripts used in this study are available at Zenodo (http://dx.doi.org/10.5281/zenodo.3590246). Raw sequences and barcodes are available at NCBI SRA database (PRJNA602211) and GenBank respectively (Table S1). Voucher specimens have been deposited at Lee Kong Chian Natural History Museum, Singapore (Table S2).
