# Supplementary material for: Transcriptome‐based target‐enrichment baits for stony corals (Cnidaria: Anthozoa: Scleractinia)
Source: Mol Ecol Resour. 2020 Mar 24;20(3):807–18. doi: 10.1111/1755-0998.13150 (PMC7468246; doi:10.1111/1755-0998.13150)
Supplement: Supplementary file 1 — Supplementary Material [file MEN-20-807-s001.pdf]

Supplemental Information for:

Transcriptome-based target-enrichment baits for stony corals (Cnidaria: Anthozoa: Scleractinia)

Randolph Z.B. Quek, Sudhanshi S. Jain, Mei Lin Neo, Greg W. Rouse, Danwei Huang

Table S1. List of COI and histone H3 sequences used in the design of barcoding baits. Bold species denote samples sequenced in this study. Asterisks indicate that reference can be found in the main text.

Species	Accession no.	Reference
**COI**		
Acanthastrea pachysepta	LN999910	Huang et al. (2016)*
Acropora palmata	AB441246	Fukami et al. (2008)*
Agaricia humilis	AB441219	Fukami et al. (2008)*
Alveopora spongiosa	AB907092	Kitano et al. (2014)*
Anacropora forbesi	AB441251	Fukami et al. (2008)*
Anomastraea irregularis	AM494869	Stefani et al. (2007)
Anthemiphyllia patera	HM018604	Kitahara et al. (2010)*
Astrea curta	HQ203286	Huang et al. (2011)*
Astroides calycularis	JQ343126	Merino-Serrais et al. (2012)
Balanophyllia (Balanophyllia) elegans	DQ445805	Hellberg (2006)
Bernardpora stutchburyi	AB907061	Kitano et al. (2014)*
Blastomussa loyae	HF954191	Benzoni et al. (2014)
Caryophyllia (Caryophyllia) ralphae	HM018617	Kitahara et al. (2010)*
Caulastraea tumida	HQ203249	Huang et al. (2011)*
Cladocora arbuscula	AB117292	Fukami et al. (2004)*
Cladopsammia eguchii	HG965317	Arrigoni et al. (2014)
Coelastrea palauensis	EU371699	Huang et al. (2008)*
Colpophyllia natans	AB117228	Fukami et al. (2004)*
Conotrochus funicolumna	HM018621	Kitahara et al. (2010)*
Coscinaraea columna	AB441210	Fukami et al. (2008)*
Craterastrea levis	HE978509	Benzoni et al. (2012)
Ctenactis crassa	LC191439	Oku et al. (2017)

**Species**	**Accession no.**	**Reference**
Ctenella chagius	AB441208	Fukami et al. (2008)*
Cyathelia axillaris	HM018622	Kitahara et al. (2010)*
Cyathotrochus pileus	HM018623	Kitahara et al. (2010)*
Cycloseris vaughani	LC191476	Oku et al. (2017)
Cynarina lacrymalis	AB117246	Fukami et al. (2004)*
Dactylotrochus cervicornis	HM018624	Kitahara et al. (2010)*
Danafungia horrida	LC191483	Oku et al. (2017)
Dasmosmilia cf. lymani	HM018625	Kitahara et al. (2010)*
Deltocyathus suluensis	HM018631	Kitahara et al. (2010)*
Dendrogyra cylindrus	AB117299	Fukami et al. (2004)*
Desmophyllum dianthus	JQ611387	Addamo et al. (2012)
Dichocoenia stokesii	AY451360	Shearer & Coffroth (2008)
Diploastrea heliopora	EU371660	Huang et al. (2008)*
Diploria labyrinthiformis	AB117224	Fukami et al. (2004)*
Dipsastraea amicorum	AB441193	Fukami et al. (2008)*
Dipsastraea favus	AB117267	Fukami et al. (2004)*
Duncanopsammia axifuga	HG965325	Arrigoni et al. (2014)
Echinophyllia echinoporoides	LT605258	Arrigoni et al. (2016a)
Echinopora pacifica	AB117261	Fukami et al. (2004)*
Eguchipsammia serpentina	HG965327	Arrigoni et al. (2014)
Enallopsammia rostrata	HM018632	Kitahara et al. (2010)*
Eusmilia fastigiata	AB117294	Fukami et al. (2004)*
Favites abdita	HQ203267	Huang et al. (2011)*
Flabellum (Flabellum) vaughani	HM018644	Kitahara et al. (2010)*
Fungiacyathus (Bathyactis) turbinolioides	HM018648	Kitahara et al. (2010)*
Galaxea fascicularis	AB441201	Fukami et al. (2008)*
Gardineria paradoxa	GQ868681	Stolarski et al. (2011)*
Gardineroseris planulata	AB441218	Fukami et al. (2008)*
Goniastrea retiformis	EU371701	Huang et al. (2008)*
Goniopora columna	AB907032	Kitano et al. (2014)*
Halomitra pileus	LC191477	Oku et al. (2017)
Heliofungia actiniformis	EU149876	Gittenberger et al. (2011)

**Species**	**Accession no.**	**Reference**
Helioseris cucullata	AB441221	Kitahara et al. (2008)*
Herpolitha limax	AB441223	Kitahara et al. (2008)*
Heteropsammia cochlea	HG965330	Arrigoni et al. (2014a)
Homophyllia australis	LN875864	Arrigoni et al. (2016b)
Homophyllia bowerbanki	LN875878	Arrigoni et al. (2016b)
Horastrea indica	AM494865	Stefani et al. (2007)
Hydnophora exesa	JQ920443	Unpublished
Isophyllia sinuosa	AB117238	Fukami et al. (2004)*
Isopora palifera	AB441248	Fukami et al. (2008)*
Javania sp.	HM018654	Kitahara et al. (2010)*
Leptastrea pruinosa	AB441196	Fukami et al. (2008)*
Leptopsammia pruvoti	HG965332	Arrigoni et al. (2014a)
Leptoria phrygia	HE654611	Arrigoni et al. (2012)
Letepsammia formosissima	GQ868684	Storlarski et al. (2011)*
Lithophyllon scabra	LC191482	Oku et al. (2017)
Lobactis scutaria	AB441224	Fukami et al. (2008)*
Madracis asanoi	HM018656	Kitahara et al. (2010)*
Madrepora oculata	HM018659	Kitahara et al. (2010)*
Manicina areolata	AB117227	Fukami et al. (2004)*
Meandrina brasiliensis	AB117297	Fukami et al. (2004)*
Merulina ampliata	AB117283	Fukami et al. (2004)*
Micromussa amakusensis	HE654643	Arrigoni et al. (2012)
Montastraea cavernosa	AB117289	Fukami et al. (2004)*
Montipora capitata	HQ246613	Forsman et al. (2010)
Moseleya latistellata	HQ203293	Huang et al. (2011)*
Mussa angulosa	AB117239	Fukami et al. (2004)*
Mussismilia hispida	AB117233	Fukami et al. (2004)*
Mycedium elephantotus	AB117388	Fukami et al. (2004)*
Mycetophyllia danaana	AB117234	Fukami et al. (2004)*
Nemenzophyllia turbida	HF954193	Benzoni et al. (2014)
Oculina diffusa	AB117293	Fukami et al. (2004)*
Orbicella faveolata	KF579908	Kirk et al. (2013)

**Species**	**Accession no.**	**Reference**
Oulastrea crispata	FJ345435	Huang et al. (2009)
Oulophyllia crispa	AB117276	Fukami et al. (2004)*
Oxypora lacera	LT605275	Arrigoni et al. (2016a)
Pachyseris speciosa	AB441222	Fukami et al. (2008)*
Paramontastraea peresi	HE654598	Arrigoni et al. (2012)
Pavona cactus	AB441216	Fukami et al. (2008)*
Pectinia paeonia	AB117386	Fukami et al. (2004)*
Phyllangia papuensis	HM018660	Kitahara et al. (2010)*
Physogyra lichtensteini	KU762014	Unpublished
Placotrochides scaphula	HM018661	Kitahara et al. (2010)*
Platygyra sinensis	HM018662	Kitahara et al. (2010)*
Plerogyra sp.	HM018663	Kitahara et al. (2010)*
Plesiastrea versipora	FR837984	Benzoni et al. (2011)
Pleuractis moluccensis	LC191471	Oku et al. (2017)
Podabacia sp.	EU149888	Gittenberger et al. (2011)
Polyphyllia talpina	EU149915	Gittenberger et al. (2011)
Porites rus	FJ423979	Forsman et al. (2009)
Pourtalosmilia anthophyllites	JQ611390	Addamo et al. (2012)
Psammocora contigua	AB441209	Fukami et al. (2008)*
Pseudodiploria strigosa	KF579902	Kirk et al. (2013)
Pseudosiderastrea tayamai	AM494866	Stefani et al. (2007)
Rhizopsammia wettsteini	HG965337	Arrigoni et al. (2014)
Rhizosmilia robusta	HM018664	Kitahara et al. (2010)*
Rhombopsammia niphada	GQ868683	Storlarski et al. (2011)*
Sandalolitha dentata	EU149914	Gittenberger et al. (2011)
Scapophyllia cylindrica	FJ345444	Huang et al. (2009)
Sclerophyllia maxima	LM993329	Arrigoni et al. (2014a)*
Scolymia cubensis	AB117236	Fukami et al. (2004)*
Seriatopora hystrix	AB441234	Fukami et al. (2008)*
Siderastrea siderea	AB441211	Fukami et al. (2008)*
Solenastrea bournoni	AB117291	Fukami et al. (2004)*
Stenocyathus vermiformis	HM018619	Kitahara et al. (2010)*

**Species**	**Accession no.**	**Reference**
Stephanocoenia intersepta	AB441228	Fukami et al. (2008)*
Stephanocyathus (Acinocyathus) spiniger	HM018665	Kitahara et al. (2010)*
Stylaraea punctata	AB907071	Kitano et al. (2014)*
Stylocoeniella guentheri	AB441225	Fukami et al. (2008)*
Trachyphyllia geoffroyi	AB117287	Fukami et al. (2004)*
Trochocyathus (Trochocyathus) rhombocolumna	HM018668	Kitahara et al. (2010)*
Tropidocyathus lessonii	HM018669	Kitahara et al. (2010)*
Truncatoflabellum sp.	HM018673	Kitahara et al. (2010)*
Tubastraea coccinea	DQ445807	Hellberg (2006)
Turbinaria peltata	AB441240	Fukami et al. (2008)*
Zoopilus echinatus	EU149916	Gittenberger et al. (2011)
**H3**		
Acanthastrea rotundoflora	LK022410	Arrigoni et al. (2014b)_
Acropora muricata	M60509	Miller et al. (1990)
Astrea curta	HQ203604	Huang et al. (2011)*
Astreopora expansa	REL0022	MN965796
Bernardpora stutchburyi	REL0029	MN965801
Caulastraea tumida	HQ203524	Huang et al. (2011)*
Coelastrea aspera	KJ666170	Huang et al. (2014a)*
Cynarina lacrymalis	LK022419	Arrigoni et al. (2014b)_
Cyphastrea microphthalma	LT575763	Arrigoni et al. (2017)
Diploastrea heliopora	HQ203531	Huang et al. (2011)*
Dipsastraea matthaii	HQ203553	Huang et al. (2011)*
Oxypora echinata	LK022415	Arrigoni et al. (2014b)_
Fimbriaphyllia ancora	REL0024	MN965798
Favites valenciennesii	HQ203611	Huang et al. (2011)*
Fungia fungites	REL0067	MN965805
Galaxea astreata	REL0020	MN965795
Goniastrea retiformis	KJ666252	Huang et al. (2014a)*
Goniopora stokesi	REL0140	MN965807
Heliofungia actiniformis	REL0153	MN965808
Herpolitha limax	REL0026	MN965800

**Species**	**Accession no.**	**Reference**
Homophyllia australis	LN875938	Arrigoni et al. (2016b)
Hydnophora microconos	HQ203594	Huang et al. (2011)*
Leptoria phrygia	HQ203597	Huang et al. (2011)*
Lobophyllia valenciennesii	LK022427	Arrigoni et al. (2014b)_
Merulina triangularis	KJ666292	Huang et al. (2014a)*
Micromussa amakusensis	LK022401	Arrigoni et al. (2014b)_
Montastraea cavernosa	HQ203601	Huang et al. (2011)*
Montipora stellata	REL0059	MN965804
Moseleya latistellata	HQ203614	Huang et al. (2011)*
Mycedium robokaki	HQ203616	Huang et al. (2011)*
Orbicella annularis	HQ203600	Huang et al. (2011)*
Oulastrea crispata	REL0016	MN965794
Oulophyllia crispa	HQ203620	Huang et al. (2011)*
Oxypora lacera	LK022417	Arrigoni et al. (2014b)_
Pachyseris speciosa	REL0025	MN965799
Paragoniastrea russelli	KJ666178	Huang et al. (2014a)*
Pavona decussata	REL00139	MN965809
Pectinia paeonia	HQ203624	Huang et al. (2011)*
Micromussa multipunctata	HQ203608	Huang et al. (2011)*
Physophyllia ayleni	HQ203622	Huang et al. (2011)*
Platygyra pini	HQ203631	Huang et al. (2011)*
Plesiastrea versipora	HQ203518	Huang et al. (2011)*
Polyphyllia talpina	REL0056	MN965803
Porites monticulosa	REL0124	MN965806
Scapophyllia cylindrica	HQ203637	Huang et al. (2011)*
Trachyphyllia geoffroyi	HQ203638	Huang et al. (2011)*
Tubastraea diaphana	REL0052	MN965802
Turbinaria mesenterina	REL0023	MN965797

Additional References

Addamo, A. M., Reimer, J. D., Taviani, M., Freiwald, A., & Machordom, A. (2012). *PLoS ONE*, *7*(11), E50215.

Arrigoni, R., Berumen, M. L., Chen, C. A., Terraneo, T., Baird, A. H., Payri, C. & Benzoni, F. (2016a). *Molecular Phylogenetics and Evolution*, *105*, 146–159.
Arrigoni, R., Benzoni, F., Huang, D., Fukami, H., Chen, C. A., … Baird, A. H. (2016b). *Contributions to Zoology*, *85*(4), 387–422.
Arrigoni, R., Kitano, Y. F., Stolarski, J., Hoeksema, B., … Benzoni, F. (2014a). *Zoological Scripta*, *43*(6), 661–688.
Arrigoni, R., Richards, Z. T., Chen, C. A., Baird, A. H., & Benzoni, F. (2014b). *Contributions to Zoology*, *83*(3), 195–215.
Arrigoni, R., Stefani, F., Pichon, M., Galli, P. & Benzoni, F. (2012). *Molecular Phylogenetics and Evolution*, *65*(1), 183–193.
Benzoni, F., Arrigoni, R., Stefani, F. & Pichon, M. (2011). *Contributions to Zoology*, *80*(4), 231–249.
Benzoni, F., Arrigoni, R., Stefani, F. & Stolarski, J. (2012). *Systematics and Biodiversity*, *10*(4), 417–433.
Benzoni, F., Arrigoni, R., Waheed, Z., Stefani, F. & Hoeksema, B. W. (2014). *Raffles Bulletin of Zoology*, *62*, 358–378.
Forsman,,Z. H., Barshis,,D. J., Hunter, C. L., & Toonen,R.J. (2009). *BMC Evolutionary Biology*, *9*, 45.
Forsman, Z. H., Concepcion, G. T., Haverkort, R. D., Shaw, R. W., Maragos, J. E. & Toonen, R. J. (2010). *PLoS ONE*, *5*(12), E15021.
Gittenberger, A., Reijnen, B. T. & Hoeksema, B. W. (2011). *Contributions to Zoology*, *80*(2), 107–132.
Hellberg, M. E. (2006). *BMC Evolutionary Biology*, *6*, 24.
Huang, D., Meier, R., Todd, P. A. & Chou, L. M. (2009). *Molecular Phylogenetics and Evolution*, *50*(1), 102–116.
Kirk, N. L., Ritson-Williams, R., Cofforth, M. A., Miller, M. W., Fogarty, N. D. & Santos, S. R. (2013). *PLoS ONE*, *8*(11), E80618.
Merino-Serrais, P. M., Casado-Amezua, P., Vicente, O. O. & Templado, J. (2012). *Graellsia*, *68*(1), 207–218.
Miller, D. J., McMillan, J., Miles, A., ten Lohuis, M. & Mahony, T. (1990). *Gene*, *93*(4), 319–320.
Oku, Y., Naruse, T. & Fukami, H. (2017). *Zoological Scripta*, *34*(3), 242–251.
Shearer, T. L. & Croffroth, M. A. (2008). *Molecular Ecology Resources*, *8*(2), 247–255.
Stefani, F., Benzoni, F., Michel, P., Cancelliere, C., & Galli, P. (2007) *Zoological Scripta*, *37*(1), 71–91.

Table S2. Collection information for species sampled in this study. Also included are catalogue numbers of specimens deposited at the Zoological Reference Collection, Lee Kong Chian Natural History Museum (Singapore).

Species	Location	GPS coordinates	Catalog numbers
**Scleractinia**			
Acropora aspera	Terumbu Raya	1.2138° N, 103.7530° E	ZRC.CNI.1418
Astreopora expansa	Sisters Islands	1.215° N, 103.8333° E	ZRC.CNI.1419
Cyphastrea serailia	Pulau Hantu	1.2223°N, 103.7499° E	ZRC.CNI.1427
Diploastrea heliopora	Raffles Lighthouse	1.1600° N, 103.7409° E	ZRC.CNI.1426
Dipsastraea maxima	Terumbu Raya	1.2138° N, 103.7530° E	ZRC.CNI.1417
Fimbriaphyllia ancora	Raffles Lighthouse	1.1600° N, 103.7409° E	ZRC.CNI.1425
Galaxea astreata	Pulau Hantu	1.2223°N, 103.7499° E	ZRC.CNI.1429
Goniastrea retiformis	Terumbu Raya	1.2138° N, 103.7530° E	ZRC.CNI.1416
Goniopora lobata	St John's Island	1.2167° N, 103.8500° E	ZRC.CNI.1430
Herpolitha limax	Raffles Lighthouse	1.1600° N, 103.7409° E	ZRC.CNI.1421
Lobophyllia radians	Raffles Lighthouse	1.1600° N, 103.7409° E	ZRC.CNI.1415
Oulastrea crispata	Pulau Hantu	1.2223°N, 103.7499° E	ZRC.CNI.1428
Pachyseris speciosa	Raffles Lighthouse	1.1600° N, 103.7409° E	ZRC.CNI.1424
Platygyra sinensis	Sisters Islands	1.215° N, 103.8333° E	ZRC.CNI.1413
Plesiastrea versipora	Kusu Island	1.2232° N, 103.8611° E	ZRC.CNI.1422
Pocillopora acuta	Sisters Islands	1.215° N, 103.8333° E	ZRC.CNI.1423
Porites lobata	Pulau Hantu	1.2223°N, 103.7499° E	ZRC.CNI.1420
Turbinaria mesenterina	Pulau Hantu	1.2223°N, 103.7499° E	ZRC.CNI.1414
**Corallimorpharia**			
Rhodactis inchoata	Sisters Islands	1.215° N, 103.8333° E	ZRC.CNI.1349
Rhodactis indosinensis	Sisters Islands	1.215° N, 103.8333° E	ZRC.CNI.1351

Table S3. Numbers of alignments, regions and baits designed based on reference genomes listed in Table 1.

Gene model	Number of alignments	Number of regions	Number of baits
Acropora digitifera	51	87	1376
Acropora tenuis	27	63	788
Fungia sp.	48	90	1118
Galaxea fasicularis	80	193	2113
Goniastrea aspera	30	73	883
Montastraea cavernosa	86	132	2041
Orbicella faveolata	28	40	617
Pocillopora damicornis	44	56	834
Porites lutea	84	156	2493
Stylophora pistillata	127	249	3656

Table S4. Statistics of Illumina sequencing reads.

Species	Raw reads	Trimmed reads (paired)	Mapped barcoding reads (reads / %)
**Scleractinia**			
Acropora aspera	12,474,167	10,449,179	–
Astreopora expansa	12,494,661	10,751,086	5,489,096 / 51.06
Cyphastrea serailia	13,036,636	10,688,227	3,995,496 / 37.38
Diploastrea heliopora	8,383,376	7,512,634	–
Dipsastraea maxima	10,317,986	9,304,707	3,972,234 / 42.69
Fimbriaphyllia ancora	11,045,605	9,045,157	3,386,460 / 37.44
Galaxea astreata	16,119,962	14,368,535	5,852,956 / 40.73
Goniastrea retiformis	12,483,736	10,839,136	–
Goniopora lobata	6,220,293	4,941,626	–
Herpolitha limax	9,627,166	8,699,061	3,017,964 / 34.69
Lobophyllia radians	9,468,583	7,769,008	–
Oulastrea crispata	8,603,319	7,625,188	2,732,530 / 35.84
Pachyseris speciosa	8,585,709	7,585,519	1,788,640 / 23.58
Platygyra sinensis	11,893,764	9,881,863	2,293,294 / 23.21
Plesiastrea versipora	8,890,660	7,077,524	1,440,808 / 20.36
Pocillopora acuta	6,597,280	5,664,946	–
Porites lobata	9,948,439	9,089,241	1,676,524 / 18.45
Turbinaria mesenterina	9,925,475	8,679,417	–
**Corallimorpharia**			
Rhodactis inchoata	7,601,449	6,538,492	–
Rhodactis indosinensis	6,825,624	5,832,989	1,290,036 / 22.12

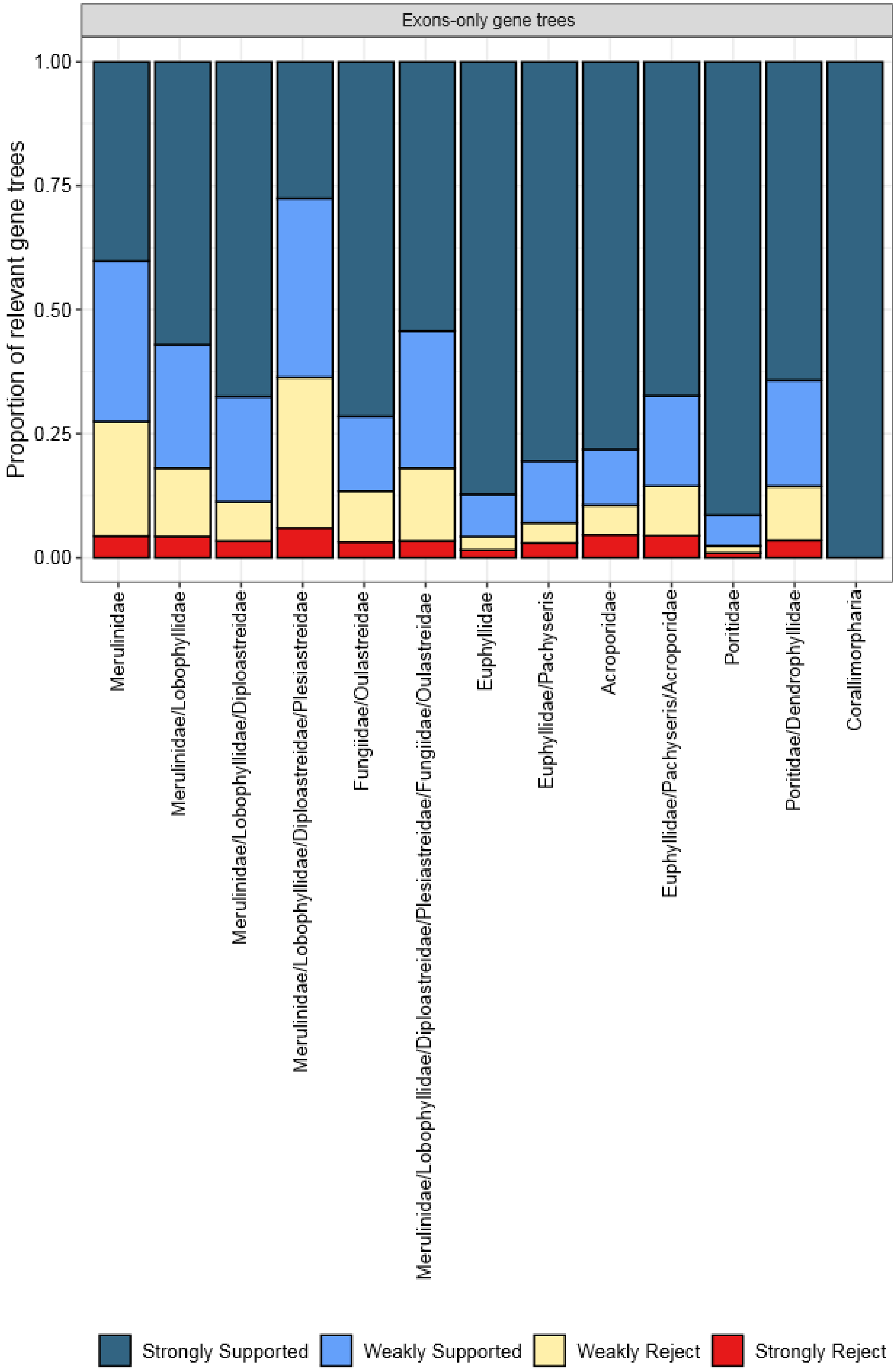

Figure S1. Proportion of maximum likelihood gene trees (RAxML) supporting family-level monophyly as inferred by DiscoVista compared to Figure 1.

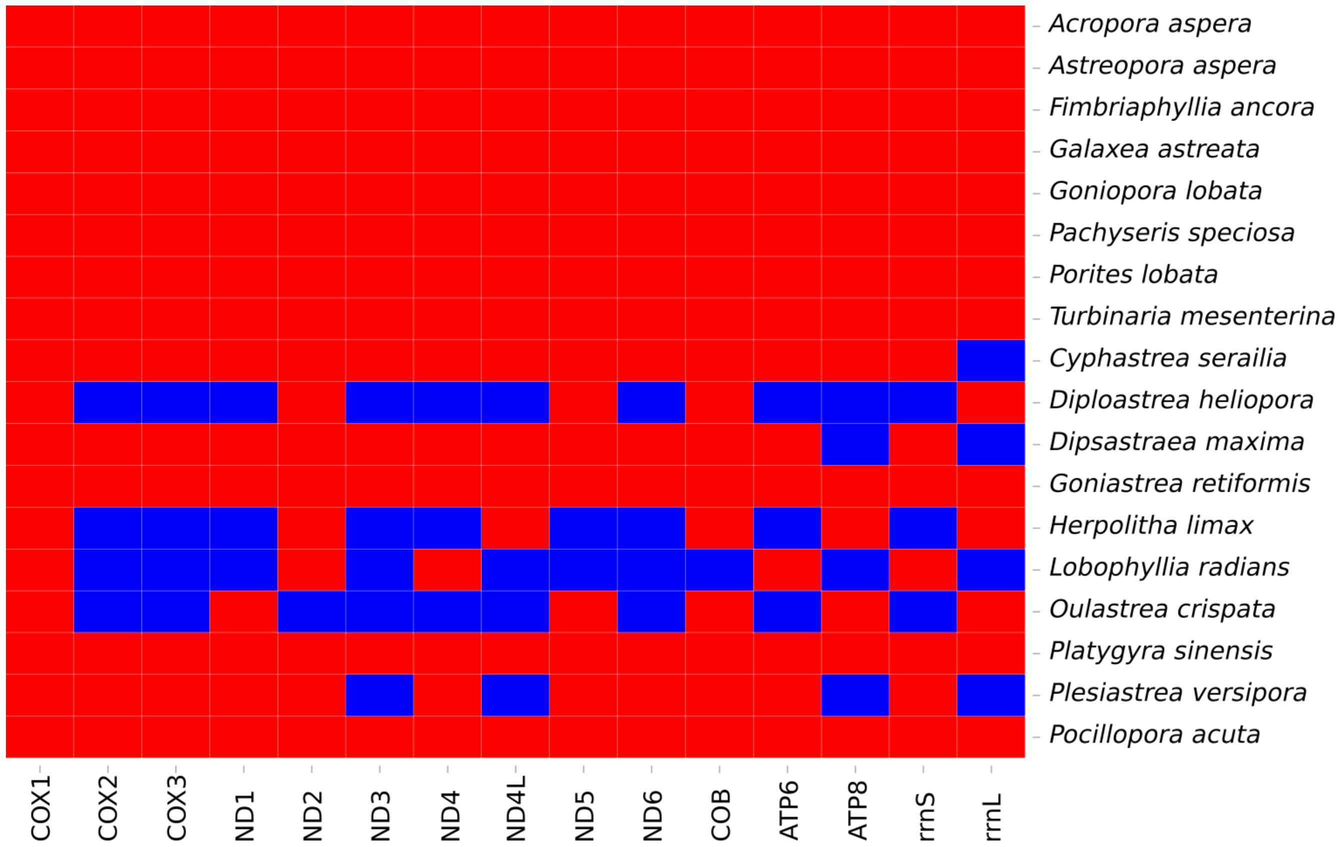

Figure S2. Coverage of mitogenomic signal recovered from assembly of trimmed reads as annotated by MITOS2 (red: present; blue: absent).

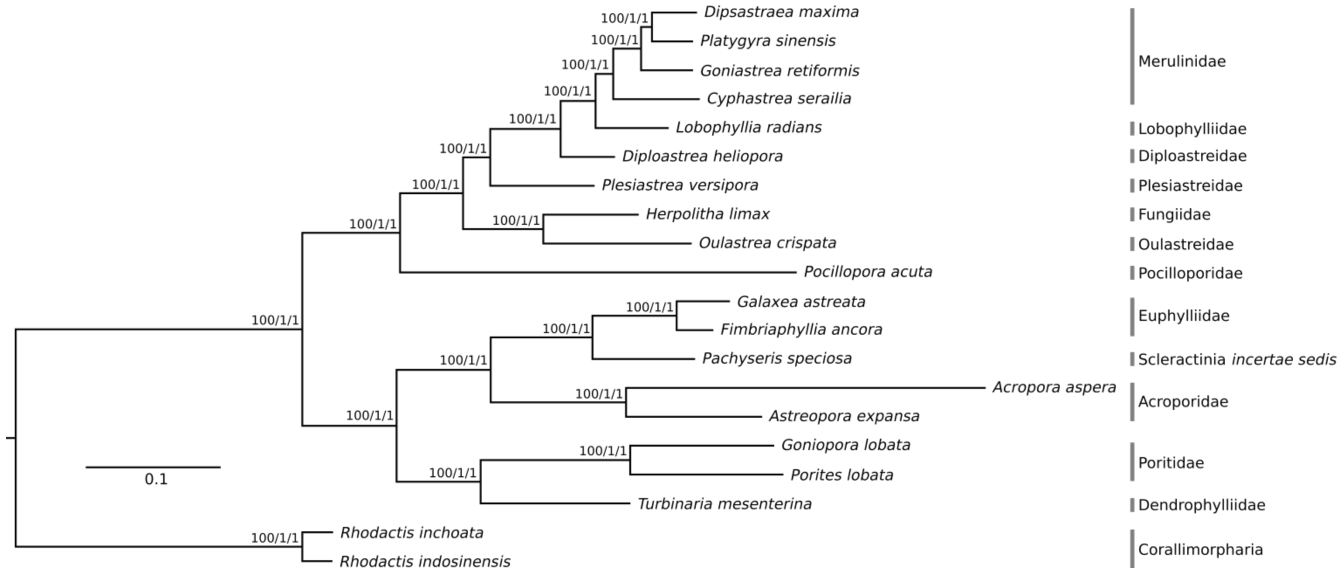

Figure S3. Maximum likelihood phylogeny of Scleractinia for exons-only dataset (minimum taxon occupancy of 3 scleractinian taxa per loci; 30.86% missing data; 452 loci over 865 exon regions; 201,137 bp) with *Rhodactis* as outgroup. Numbers on nodes represent bootstrap values (RAxML) / posterior probability (ExaBayes) / posterior probability (ASTRAL).
